# Online Communication Between Doctors and Patients in Europe: Status and Perspectives

**DOI:** 10.2196/jmir.1281

**Published:** 2010-06-15

**Authors:** Silvina Santana, Berthold Lausen, Maria Bujnowska-Fedak, Catherine Chronaki, Per Egil Kummervold, Janne Rasmussen, Tove Sorensen

**Affiliations:** ^7^Norwegian Centre for Integrated Care and TelemedicineUniversity Hospital of North-NorwayTromsøNorway; ^6^Odense University HospitalOdenseDenmark; ^5^Institute of Clinical MedicineUniversity of Tromsø and Norwegian Centre for Integrated Care and TelemedicineUniversity Hospital of North-NorwayTromsøNorway; ^4^Institute of Computer ScienceFoundation for Research and Technology-HellasHeraklionGreece; ^3^Department of Family MedicineWrocław Medical UniversityWrocławPoland; ^2^Department of Mathematical SciencesUniversity of EssexColchesterUnited Kingdom; ^1^Institute of Electronics Engineering and Telematic of Aveiro and Department of Economics, Management and Industrial EngineeringUniversity of AveiroAveiroPortugal

**Keywords:** Online physician-patient interaction, email communication, online prescription ordering, scheduling appointments online, eHealth services utilization and trends, Internet, Europe, survey, logistic regression analysis

## Abstract

**Background:**

Use of the Internet for health purposes is steadily increasing in Europe, while the eHealth market is still a niche. Online communication between doctor and patient is one aspect of eHealth with potentially great impact on the use of health systems, patient-doctor roles and relations and individuals’ health. Monitoring and understanding practices, trends, and expectations in this area is important, as it may bring invaluable knowledge to all stakeholders, in the Health 2.0 era.

**Objective:**

Our two main goals were: (1) to investigate use of the Internet and changes in expectations about future use for particular aspects of communication with a known doctor (obtaining a prescription, scheduling an appointment, or asking a particular health question), and (2) to investigate how important the provision of email and Web services to communicate with the physician is when choosing a new doctor for a first time face-to-face appointment. The data come from the second survey of the eHealth Trends study, which addressed trends and perspectives of health-related Internet use in Europe. This study builds on previous work that established levels of generic use of the Internet for self-help activities, ordering medicine or other health products, interacting with a Web doctor/unknown health professional, and communicating with a family doctor or other known health professional.

**Methods:**

A representative sample of citizens from seven European countries was surveyed (n = 7022) in April and May of 2007 through computer-assisted telephone interviews (CATI). Respondents were questioned about their use of the Internet to obtain a prescription, schedule an appointment, or ask a health professional about a particular health question. They were also asked what their expectations were regarding future use of the Internet for health-related matters. In a more pragmatic approach to the subject, they were asked about the perceived importance when choosing a new doctor of the possibility of using email and the Web to communicate with that physician. Logistic regression analysis was used to draw the profiles of users of related eHealth services in Europe among the population in general and in the subgroup of those who use the Internet for health-related matters. Changes from 2005 to 2007 were computed using data from the first eHealth Trends survey (October and November 2005, n = 7934).

**Results:**

In 2007, an estimated 1.8% (95% confidence interval [CI], 1.5 - 2.1) of the population in these countries had used the Internet to request or renew a prescription; 3.2% (95% CI 2.8 - 3.6) had used the Internet to schedule an appointment; and 2.5% (95% CI 2.2 - 2.9) had used the Internet to ask a particular health question. This represents estimated increases of 0.9% (95% CI 0.5 - 1.3), 1.7% (95% CI 1.2 - 2.2), and 1.4% (95% CI 0.9 - 1.8). An estimated 18.0% (95% CI 17.1 - 18.9) of the populations of these countries expected that in the near future they would have consultations with health professionals online, and 25.4% (95% CI 24.4 - 26.3) expected that in the near future they would be able to schedule an appointment online. Among those using the Internet for health-related purposes, on average more than 4 in 10 people considered the provision of these eHealth services to be important when choosing a new doctor.

**Conclusions:**

Use of the Internet to communicate with a known health professional is still rare in Europe. Legal context, health policy issues, and technical conditions prevailing in different countries might be playing a major role in the situation. Interest in associated eHealth services is high among citizens and likely to increase.

## Introduction

Quality health care depends on successful communication between health professionals and patients [1]. As the use of Web tools becomes more pervasive in health and medicine as represented by the concepts Health 2.0 [2] and Medicine 2.0 [3], and patients become more empowered, all parties need to adjust to a new form of participatory health care. These new environments are likely to promote more personalized health care, increased collaboration, and better health education. Expected outcomes are not only improved health but also more efficiency in the use of scarce resources, improved trust between stakeholders, and greater convenience [3], the essence of quality health care. Prior work suggests that online communication tools such as the Web and email can play important roles in enhancing access to health care and health information, in facilitating clinical management [4], and in increasing the effectiveness of practice administration. Such tools might even play roles in reducing health system expenditure [5] and in increasing overall efficiency [6]. However, a number of barriers and risks have also been identified [7-15]. Evidence from recent fieldwork is mixed, probably because assessment has involved varying methodologies, settings, systems, and perspectives [5,6,13,16-19].

Some studies have indicated that demand for online communication is strong among patients [20,21] and that, among Internet users, willingness to pay for Web portal services does not appear to vary significantly with age [22]. In one study of pediatric primary care, parents were particularly enthusiastic about the possibility of communicating online with their child’s physician [23], stating that the ability to communicate online might be a reason to choose a particular pediatrician, even though the majority said they were unwilling to pay for such access [24]. In another study, older patients responded that they would like to use email to communicate with their physicians [25].

Regarding level of actual use, some studies of providers and consumers have found that online correspondence among patients and physicians, both solicited and unsolicited, has increased dramatically, while other studies have found this type of communication to be more limited. In New Zealand, for example, 68% of the 80 general practitioners interviewed in one study had never used email to communicate with their patients, and only 4% had used it regularly [26]. An investigation conducted in the east of Scotland found similar levels of use [27]. On the other hand, a cross-sectional study involving all physicians at the Finnish Student Health Service found that 79% of these physicians use email to communicate with patients [28] the same level reported by other studies of email communication in similar settings [17]. Studies involving the general population have reported much lower but also disparate levels of use. An online survey conducted in the United States in 2006, found that while only up to 4% of adults use or have access to online services for communicating with their physicians, most would like to communicate with their physicians in this way. In fact, the majority stated that the availability of online services would influence their choice of health care provider to some extent [29]. Meanwhile, results from the Health Information National Trends Surveys, HINTS 2003 and HINTS 2005, [30] showed that in 2003, 7% of American Internet users had used email or the Internet to communicate with a physician or a physician’s office in the past 12 months, a proportion that had increased significantly to 10% by 2005. In Europe, the World Health Organization (WHO)/European Survey on eHealth Consumer Trends (eHealth Trends) [31] found that the estimated percentage of the population that had approached a family doctor or other known health professional through the Internet, even if only to read their website, had increased from 3.6% in 2005 to 6.9% in 2007, while the percentage of those interacting with a Web doctor or health professional they had never met increased from 8.2% to 11.1% over the same period. Results from a 2007 online survey of Dutch primary care patients with chronic complaints [32], a relevant target group for e-consultations, revealed that 90% had had no prior experience with such a service.

By the time of the second eHealth Trends survey, European countries had established priorities and strategies for eHealth [33]. However, the conditions reported to have been in place in the seven countries that participated in the eHealth Trends survey were very different. In Denmark, several initiatives were in place that aimed at the development of common standards, concepts, and classifications; good integration between electronic health records (EHR) and other health information systems; and the implementation of an Internet-based health care data network. The Danish public national health portal, Sundhed.dk, had been launched in 2003 [34].

In Germany, the two pillars of modernization identified were the establishment of an information and communication technology (ICT) infrastructure and the implementation of a private electronic patient record. The latter, to be introduced in four stages, would allow the provision of administrative data and transmission of electronic prescriptions, among other things [35]. With respect to a legal and regulatory framework, national legislation addressing telemedicine and eHealth service provision was in place. The German Medical Association's professional code of conduct (*Berufsordnung der Ärztekammer*) restricted the exchange of health-related email between doctor and patient to situations where there had been previous face-to-face contact.

In Greece, plans for the period 2006 to 2007 aimed at strengthening standardization and communication infrastructures and preparing the path for national integration by 2015. The plan was to do this through spearheading pilot projects linked to Europe-wide efforts with health insurance cards, e-prescription, and telemedicine [36].

In Latvia, development of telemedicine and provision of health care services online were two of the priorities defined, but by April 2007 the assessment of progress achieved so far was considered irrelevant given that the implementation plan was to have been ready at the end of 2006. As of 2007, there was no legal framework specific for eHealth or telemedicine practice available [37].

In Norway, the implementation of a national eGovernment information portal serving all sectors, including health, that might give access to e-prescription, the implementation of eResept (for electronic communication of prescription information), and the clarification of responsibility, rules, guidelines, and costs in connection with telemedicine consultations were among future activities to be developed. Legislative research started during spring 2006 to determine ways in which existing legislation was hindering progress in eHealth [38].

In Poland, development of electronic communication in health care, telemedicine services, and a central health care portal were some of the strategic targets in the national eHealth roadmap, but by 2007 much seemed to be still at the conceptual phase, and no specific legal framework for eHealth was available [39].

In Portugal, the promotion of telemedicine initiatives and development of e-prescription functionalities were some of the future activities envisaged [40]. There was no legal framework specific for eHealth or telemedicine practice, but online interaction in general involving personal data exchange and diffusion, such as eHealth services, come under very strict legislation that discourages online communication with patients in a clinical setting, especially in private and small practices.

Technical and legal differences in European countries exist together with spreading use of the Internet and email in Europe and the increased potential of these technologies to change the boundaries of communication within medical practice as well as several dimensions of the patient-physician relationship. Therefore, there is an urgent need to investigate how such services are being used, appraised, and valuated by European citizens.

For this paper, we used data of the second eHealth Trends survey that were not analyzed in previous work [31]. We used these data to investigate in the seven participating countries use of the Internet and email to interact with known health professionals for specific online services. First, we report on current levels of use of the Internet to obtain a prescription, schedule an appointment, or to ask a particular health question. We also report on changes in Internet use and expectations about future use that have occurred over the 18 month period following the first eHealth survey administered in 2005 in the seven countries. In addition, we report the importance of the availability of email and Web services for communicating with the physician when choosing a doctor for a first-time face-to-face appointment. Finally, using the results of logistic regression analysis of the data, we draw profiles of the potential consumers of related eHealth services. The discussion focuses on implications for citizens, health care providers, policy makers, and other stakeholders across Europe.

## Methods

### Participants and Procedures

Residents of Denmark, Germany, Greece, Latvia, Norway, Poland, and Portugal participated in the study. The questionnaire was administered through computer-assisted telephone interviews (CATI). The survey was conducted in April and May 2007 to reach a target of a representative sample of 1000 complete questionnaires in each participating country. Because the 2005 eHealth Trends survey was found to be skewed for some age groups, the 2007 survey used quotas based on census data. Six groups were defined based on age and gender specific to each country, and random digit dialing within strata was used to ensure a randomized representative sample (for more details see [31]). In total, 7022 questionnaires were completed, corresponding to an average response rate of 36% of the 22,867 individuals contacted in the seven countries (for more details see [31]).

Multimedia Appendix 1 shows the frequencies of respondents across age groups by gender and the sociodemographic variables included in the 2007 questionnaire. For comparison, we present the frequencies and percentages of the subjective health status of the European Social Survey (ESS) (September 2006 through March 2007). The eHealth Trends survey used the same question to report subjective health as the ESS, but the ESS used face-to-face interviews at home, with a reported response rate above 60%. The ESS did not cover Greece and Latvia. For the other five countries both studies obtained similar patterns. 

### Measures

The level of Internet use to obtain a prescription, schedule an appointment, or ask a particular health question was assessed by Question A: “In which connection and for what purpose have you approached your family doctor, specialist, or other health professional(s) via the Internet?” The various nonexclusive possibilities included: “request or renew prescription via email or web,” “schedule an appointment,” and “ask a particular health question.” Expectations about future use were appraised by Question B: “Given that you were provided the possibility, state how likely it is that you will do the following during the next year: (1) have consultations with a health professional online and (2) make, cancel, or change an appointment with your family doctor, specialist, or other health professionals online?” The response categories ranged from 1 (“unlikely”) to 5 (“very likely”). The importance attributed by European residents to the provision of email and Web services to communicate with the physician when choosing a new doctor was measured by Question C: “If you were to find a new doctor, state the importance of the following factors for your decision: (1) the possibility of requesting or renewing prescriptions via email or Web, (2) the possibility of scheduling or changing an appointment online, and (3) the possibility of communicating by email.” The response categories ranged from 1 (“not important”) to 5 (“important”).

The focus on factors influencing the choice of a new doctor is relevant to our study. In this way, aspects such as previous experience with a health care provider, either at a clinical or administrative level that might lead to dependence and loyalty, are not considered, and we intended the question to address respondents’ perceptions, attitudes, and expectations created through other mechanisms, such as their own experience with online services, knowledge about the experiences of others, or exposure to related information from mass media.

The questionnaire was designed in English and translated to the language or languages of the participant countries by means of a dual focus method, which strives for conceptual correspondence in addition to equivalence in wording and grammar [41].

### Data Analysis

Tables 1 to 3 and Multimedia Appendices 2 to 6 provide 95% confidence intervals (CI) derived from Gaussian approximations of the distribution of the sum of strata frequencies or sum of ratios of strata frequencies. *P* values of two-sided tests are not given. For each test, significant test results are reported when the null is not inside the 95% interval. Differences (2007 minus 2005) were computed using poststratified data of the first eHealth Trends survey (October to November 2005) in the analyses (Tables 1 to 3 and Multimedia Appendices 2 to 6). Poststratified weighting of the 2005 distribution was defined by weights based on the 2007 distribution that used six age groups (15-25, 26-35, 36-45, 46-55, 56-65 and 66-80 years) by gender. This was done in order to separate real effects from minor changes introduced by sample construction (for more details see [31]). Binary outcomes of question C were analyzed as dependent variables by logistic regression on demographic, socioeconomic, and health variables. We fitted full models including all independent variables reported in Table 4 and Multimedia Appendix 7. Interaction terms were not fitted. We investigated two different groups: the total sample, which represented the general population, and a subsample of respondents who reported that they had used the Internet for health-related matters in the past. For each factor level, the odds ratio (OR) and 95 % confidence interval of the odds ratio were reported. Factors were tested with type II hypotheses (function Anova, R package: car version 1.2-7) (R Foundation for Statistical Computing, Vienna, Austria). Type-II hypotheses test each term after all others but ignore the term’s higher-order relatives. No variables had more than 5% missing data.

All analyses were performed with SPSS version 16.0 (SPSS Inc, Chicago, IL, USA) and R version 2.8.1 (R Foundation for Statistical Computing, Vienna, Austria) [42].

## Results

### Patterns of Use of the Internet to Communicate With Known Health Professionals

Table 1 shows the prevalence of Internet use in 2007 to request or renew a prescription via email or the Web, schedule an appointment, or ask a particular health question, as well as the changes in the period between the two surveys in the seven European countries (for detailed results for each country, see Multimedia Appendices 2 to 4).

**Table 1:** Patterns of use of the Internet to communicate with known health professional

	2007		Change From 2005 to 2007	
	General Population	Health-related Internet Users	General Population	Health-related Internet Users
	Frequency Mean % (CI)^a^	Frequency Mean % (CI)^a^	Mean % (CI)^a^	Mean % (CI)^a^
Request or renew prescription	130	130		
	1.8 (1.5 - 2.1)	2.8 (2.4 - 3.3)	*+0.9 (0.5 - 1.3)*	*+1 (0.4 - 1.7)*
Schedule an appointment	226	226		
	3.2 (2.8 - 3.6)	5.4 (4.7 - 6.1)	*+1.7 (1.2 - 2.2)*	*+2.3 (1.2 - 3.3)*
Ask a particular health question	178	178		
	2.5 (2.2 - 2.9)	4.7 (4.0 - 5.4)	*+1.4 (0.9 - 1.8)*	*+2.2 (1.3 - 3.1)*

^a^ 95% confidence intervals (CI); differences appear in italics when significantly different from 0 at the 5% level

The highest levels of Internet use were found in Denmark where, in 2007, an estimated 7.4% (95% CI 5.8 - 9.1) of the population reported having approached a family doctor, specialist, or other health professional via email or the Web to request or renew a prescription, 9.9% (95% CI 8.0 - 11.8) to schedule an appointment, and 6.7% (95% CI 5.1 - 8.2) to ask particular health questions. The lowest levels of Internet use in 2007 were found in Portugal, with an estimated use for these purposes of 0%, 0.4% (95% CI 0.1 - 0.7), and 0.7% (95% CI 0.3 - 1.1), respectively. The subgroup of health-related Internet users, who were experienced in looking for information on health matters on the Internet, were more active in using eHealth services than the population in general.

### Expectations Regarding Future Use of eHealth Services

Table 2 shows the estimated percentages of European citizens and of European health-related Internet users expecting to have consultations with a health professional online or schedule an appointment with a family doctor, specialist, or other health professional online in the near future in 2007, as well as the mean changes in their expectations from 2005 to 2007 in the seven countries. Detailed results for each country are shown in Multimedia Appendices 5 and 6.

**Table 2:** Expectations regarding future use of eHealth services

	2007		Change From 2005 to 2007	
	General Population	Health-related Internet Users	General Population	Health-related Internet Users
	Frequency Mean % (CI)^a^	Frequency Mean % (CI)^a^	Mean % (CI)^a^	Mean % (CI)^a^
Have consultations with health professional	1264	876		
	18.0 (17.1 - 18.9)	24.1 (22.7 - 25.5)	*-2.5 (-3.7 to -1.2)*	*-4.9 (-7.1 to -2.7)*
Schedule or change an appointment online	1783	1244		
	25.4 (24.4 - 26.3)	32.5 (31.0 - 34.0)	+0.8 (-0.6 to 2.2)	-0.4 (-2.6 to 1.9)

^a^ 95% confidence intervals (CI); differences appear in italics when significantly different from 0 at the 5% level

Among the general population, results showed a decrease in the average percentage of citizens stating they were likely to have consultations with a health professional online and a nonsignificant increase in the average percentage of citizens that believed they were likely to schedule an appointment online in the near future. In the subsample of health-related Internet users, average percentages decreased in the two situations. Denmark stands out as the country where citizens’ expectations had increased most since 2005. In 2007, an estimated 26.2% (95% CI 23.5 - 28.8) of Danes stated they were very likely to have consultations with a health professional online in the year following the survey, and an estimated 36.8% (95% CI 33.9 - 39.7) stated they were very likely to schedule or change an appointment online. This corresponds to estimated mean increases of 5.3% (95% CI 1.5 - 9.0) and 7.3% (95% CI 3.3 - 11.3) respectively from 2005 to 2007.

On the other hand in Poland, citizens’ expectations had decreased the most since 2005. In 2007, an estimated 25% (95% CI 22.3 - 27.7) of Poles stated they were very likely to have consultations with a health professional online in the year following the survey, representing a decrease of 13.3% (95% CI -17.3 to -9.4) from 2005, and 27.3% (95% CI 24.6 - 30.0) affirmed they were very likely to schedule or change an appointment online, a decrease of 8.7% (95% CI -12.7 to -4.7) since 2005. In 2005, however, Polish citizens’ expectations were highest among the European countries studied. In most of the seven countries (as shown in Multimedia Appendices 5 and 6), increases were more pronounced among the general population, while decreases were more pronounced among those using the Internet for health-related purposes. The exception was Latvia, where the average percentage of health-related Internet users expecting to be able to schedule an appointment with a health professional online had increased by 4.9% (95% CI -1.1 to 11.0) from 2005 to 2007.

### Importance of eHealth Services When Choosing a New Doctor

Table 3 shows the estimated mean percentages of European citizens and of European health-related Internet users that rated the importance of various eHealth services (using email or the Web) when choosing a new doctor at 4 or 5 (important) on a scale of 5 points in 2005 and 2007 and the mean changes from 2005 to 2007.

**Table 3:** Importance of eHealth services when choosing a new doctor in 2007 and changes from 2005 to 2007

	2007		Change From 2005 to 2007	
	General Population	Health-related Internet Users	General Population	Health-related Internet Users
	Frequency Mean % (CI)^a^	Frequency Mean % (CI)^a^	Mean % (CI)^a^	Mean % (CI)^a^
The possibility of requesting or renewing prescriptions	1996	1379		
	28.4 (27.4 - 29.4)	37.3 (35.7 - 38.8)	*+2.6 (1.2 - 4.0)*	+1.0 (-1.3 to 3.3)
The possibility of scheduling or changing an appointment	2690	1886		
	38.3 (37.2 - 39.4)	52.0 (50.4 - 53.6)	*+3.2 (1.8 - 4.7)*	+1.3 (-1.1 to 3.7)
The possibility of communicating by email	2644	1858		
	37.7 (36.6 - 38.7)	51.4 (49.8 - 53.1)	*+3.2 (1.7 - 4.7)*	-0.5 (-2.9 to 2.0)
All three services	1237	933		
	17.6 (16.7 - 18.5)	25.2 (23.8 - 26.6)	*+1.3 (0.1 - 2.5)*	+0.2 (-1.9 to 2.2)
At least one service	3702	2482		
	52.7 (51.6 - 53.8)	68.6 (67.1 - 70.1)	*+4.6 (3.0 - 6.1)*	+0.2 (-2.1 to 2.4)

^a^ 95% confidence intervals (CI); differences appear in italics when significantly different from 0 at the 5% level

Scheduling or changing an appointment online and communicating with a health professional by email are the most appealing services. In 2007, the former was rated as important by 38.3% (95% CI 37.2 - 39.4) of citizens, corresponding to 2690 individuals and representing a mean change of 3.2% (95% CI 1.8 - 4.7) from 2005. The possibility of communicating by email was valued by a mean percentage of 37.7% (95% CI 36.6 - 38.7) citizens in 2007, corresponding to 2644 individuals and representing a mean change of 3.2% (1.7 - 4.7) from 2005, while the possibility of requesting or renewing prescriptions via email or the Web was quoted as an important factor in their decision by 28.4% (95% CI 27.4 - 29.4) of citizens, corresponding to 1996 individuals and representing a mean change of 2.6% (95% CI 1.2 - 4.0) from 2005. Changes are less significant among health-related Internet users. On average in 2007, half of the population reported that the possibility of having access to at least one eHealth service was important in their decision, while among health-related Internet users the number rises to 7 of every 10 respondents.

### Characteristics of Survey Participants who Value eHealth Services When Choosing a New Doctor

Table 4 reports the results of logistic regression analyses that examined relationships between demographic, socioeconomic, and health variables, and the outcomes of the question concerning the importance of eHealth services when choosing a new doctor in the subsample of Internet users for matters related to health or illness. (For detailed results with respect to the general population see Multimedia Appendix 7.)

The estimated odds ratios indicated that those health-related Internet users who appreciated most the possibility of requesting or renewing a prescription via email or the Web when choosing a new doctor were under 25 (OR = 1.97, 95% CI 1.27 - 3.06), had secondary school education (OR = 1.37, 95% CI 1.10 - 1.71) and probably lived in main cities (OR = 1.39, 95% CI 1.08 - 1.78). Among the general population, young, well-educated (OR = 1.68, 95% CI 1.43 - 1.97) working people (OR = 1.27, 95% CI 1.08 - 1.46) no older than 25 (OR = 3.00, 95% CI 2.28 - 3.95) were most interested in this eService. In general, odds ratios for age and education are lower in the subsample, reflecting smaller differences between the groups. In the subsample of health-related Internet users, those valuing most the possibility of scheduling an appointment online were aged 26 to 35 (OR = 2.75, 95% CI 1.81 - 4.19), had no disability or diagnosed illness (OR = 0.67, 95% CI 0.54 - 0.84), and lived in main cities (OR = 1.66, 95% CI 1.30 - 2.11). Multimedia Appendix 7 shows that among the general population young people up to 25 years old (OR = 4.95, 95% CI 3.78 - 6.48) still in education (OR = 1.34, 95% CI 1.07 - 1.68), having some higher education (OR = 1.61, 95% CI 1.38 - 1.87), and living in main cities (OR = 1.30, 95% CI 1.09 - 1.55) were those most frequently interested in the service. The possibility of communicating with a health professional by email seemed particularly appealing to well-educated (OR = 1.93, 95% CI 1.66 - 2.25) working citizens (OR = 1.41, 95% CI 1.22 - 1.62) up to 25 years old (OR = 3.54, 95% CI 2.72 - 4.60). In the restricted sample, we observed significant results for those with secondary education (OR = 1.58, 95% CI 1.28 - 1.96).

**Table 4:** Characteristics of health-related Internet users who value eHealth services when choosing a new doctor in 2007

		Request or Renew Prescriptions			Schedule or Change Appointments Online			Communicate by email with Health Professionals		
		Odds Ratio	95% CI	*P* value	Odds Ratio	95% CI	*P* value	Odds Ratio	95% CI	*P* value
**Gender**										
	Female	1.08	0.94 - 1.24	.29	1.00	0.88 - 1.14	.99	0.95	0.86 - 1.09	.49
	Male	1			1			1		
**Age**				.005			< .001			.17
	15-25	1.97	1.27 - 3.06	.003	2.68	1.73 - 4.16	< .001	1.43	0.95 - 2.16	.09
	26-35	1.85	1.21 - 2.81	.004	2.75	1.81 - 4.19	< .001	1.50	1.01 - 2.21	.04
	36-45	1.76	1.14 - 2.70	.01	2.58	1.68 - 3.95	< .001	1.53	1.02 - 2.28	.04
	46-55	1.36	0.89 - 2.10	.16	2.19	1.43 - 3.35	< .001	1.22	0.82 - 1.81	.33
	56-65	1.44	0.93 - 2.21	.10	2.23	1.45 - 3.42	< .001	1.32	0.89 - 1.97	.18
	66-80	1			1			1
**Education**				.01			.10			< .001
	Higher education	1.14	0.93 - 1.40	.21	1.24	1.02 - 1.51	.0**3**	1.34	1.10 - 1.63	.004
	A-Level	1.37	1.10 - 1.71	.006	1.22	0.98 - 1.51	.07	1.58	1.28 - 1.96	< .001
	below A-Level	1			1			1		
**Children at home ** **( < 18)**		1.00	0.86 - 1.17	.96	1.06	0.91 - 1.23	.44	1.00	0.86 - 1.16	.99
	No children at home	1			1			1		
**Place of residence**				.03			< .001			.21
	City	1.39	1.08 - 1.78	.01	1.66	1.30 - 2.11	< .001	1.22	0.96 - 1.54	.11
	Minor city	1.20	0.93 - 1.54	.17	1.37	1.07 - 1.75	.01	1.23	0.97 - 1.57	.09
	Village	1.14	0.88 - 1.48	.33	1.45	1.13 - 1.86	.004	1.31	1.02 - 1.68	.04
	Rural/remote location	1			1			1		
**Work situation**				.31			.22			.24
	Student	0.84	0.63 - 1.12	.23	1.10	0.83 - 1.45	.52	1.14	0.86 - 1.50	.37
	Working	1.02	0.83 - 1.25	.85	1.19	0.98 - 1.45	.08	1.19	0.97 - 1.44	.09
	Not working	1			1			1		
**With diagnosed illness**		0.85	0.68 - 1.07	.16	0.67	0.54 - 0.84	< .001	0.82	0.66 - 1.01	.07
	No	1			1			1		
**Relative with** **diagnosed illness**		0.90	0.76 - 1.05	.17	0.86	0.74 - 1.00	.05	0.94	0.80 - 1.09	.39
	No	1			1			1		
**Subjective health status**				.11			.62			.55
	Good	0.84	0.56 - 1.26	.40	0.94	0.63 - 1.40	.76	0.84	0.57 - 1.24	.39
	Fair	1.01	0.67 - 1.52	.97	1.02	0.68 - 1.53	.91	0.80	0.54 - 1.20	.28
	Bad	1			1			1		

## Discussion

Use of the Internet to communicate with a known health professional is still rare in Europe, but interest in using it is high and likely to increase. Denmark is the only country in which consistent increases were found in all the variables under analysis in the 18 months between the two surveys.

### Patterns of Internet Use to Communicate With Known Health Professionals

As expected, the estimated level of use of the Internet to request or renew prescriptions via email or the Web, schedule an appointment, or ask a known health professional a particular health question is still very low in the seven countries. Although not directly comparable, these results are in line with other reports of the use of these means to communicate with a doctor or a doctor’s practice in the United States [30]. One question remains: were the reasons for such low use the same in the two settings? In Europe, technologies that enable electronic storage of administrative patient data and of medical patient data seem to be available [43], at least in countries like Denmark, Norway, the United Kingdom, Sweden, the Netherlands, and Germany, although in Germany these technologies are not as available as in the other countries. However, use of email to communicate with patients may be in contravention of health authority policy. Benchmarking studies have confirmed that using the Internet or electronic health networks to email patients about health or even administrative issues is very rare (around 3%) in general practitioners’ practices throughout Europe [43], Denmark (at about 60%) being a clear exception.

In the United States, as early as 2001, the Institute of Medicine asserted that “patients should receive care whenever they need it and in many forms, not just face-to-face visits” and that “access to care should be provided over the Internet, by telephone, and by other means [44].” Meanwhile, the level of adoption of other health information technology by the health sector is still low and will likely remain slow unless significant financial incentives are made available [45,46]. Reported experiences with Web messaging have been promoted and financially supported by some health plans in the United States [5]. Many aspects of electronic communication related to reimbursement, legal issues, trust, and security remain unclear and need to be addressed.

The change in use during the 18 months between the two eHealth Trends surveys in Europe is statistically significant, even though certainly not impressive. However, the real relevance of this change must be analyzed in the light of the legal and regulatory environment prevailing in Europe and the policies governing health care delivery in the participating countries, as well as the technological achievements in each country. Despite the stated intentions [47], so far little seems to have changed in most of the countries studied. In Greece, for example, the legal framework for e-prescription and reimbursement is still incomplete.

According to a federal government report issued in Germany in April 2009 [48], the electronic health card is being piloted there, and electronic prescription has not been implemented so far. While email is available for interaction with most practitioners in Germany, it is not normally used for consultation but for administrative requests only, while the directive from the German Medical Association regarding email communication between doctor and patient remains [49].

In Poland, there are still no specific policies or legal regulations that could encourage online or even telephone medical consultations. Currently, basic online eHealth services are rare and there are few services offering online interaction with general practitioners. In 2004 the Ministry of Health issued an internal document called “Poland: eHealth Strategy for 2004-2006,” which stated that strategies for eHealth are part of the broader effort focused on development of the information society in Poland [47]. This might be the reason why the number of Poles expecting to use eHealth services in the year following the survey was the highest among the European countries studied in the 2005 survey. However, in spite of many promises and assurances from the government, the situation has not changed during the last 2 years and it still lacks regulation. Such regulations could solve the legal problems associated with eHealth consultations, enabling eHealth services to be officially implemented and reimbursed. On the other hand, attempts to implement and develop a registry of health services based on the use of electronic health insurance cards have been underway for the last ten years [47], but there is still no common and functional EHR system. These situations are probably part of the explanation for such a large decrease in citizens’ expectations in Poland in the 18 months between the two surveys.

In Norway, direct communication between patients and health professionals is limited to making appointments. It is illegal in Norway to communicate about personal health matters (ie, providing personally identifiable information) via email unless a special encrypted service is used. So far, only a minority of general practitioners’ (GP) offices in Norway offer this service.

In Portugal, the implementation of a national EHR system is currently under public discussion, while specific legislation for eHealth is still missing.

### Expectations Regarding Future Use of eHealth Services

Overall, the citizens of the seven countries surveyed seemed to have had low expectations regarding the likelihood of having consultations with health professionals or being able to schedule appointments online in the period from 2005 to 2007. A study conducted in the United Kingdom in 2003 [50] concluded at the time that “patients simply doubted whether it was possible for the National Health Service and technology to cope with patients ordering prescriptions, emailing GPs, and booking appointments online.” Therefore, further work is needed to assess how our results should be interpreted, whether as a sign of doubt regarding the capacity of health systems and technology to handle such demands, as a reaction to the current level of provision, or as a barometer of the need and intention to use. Meanwhile, it is almost impossible not to question the extent to which the delay in implementation of the “information society” in many European countries is lowering the expectations of their citizens and cooling down enthusiasm for these new services. This may be the reason why the decrease in expectations concerning the use of eHealth services in the near future is higher among those experienced in using the Internet for health-related matters.

### Importance of eHealth Services When Choosing a New Doctor

Nevertheless, the percentage of citizens that considered the availability of such eHealth services to be important when choosing a new doctor increased significantly by around 3% from 2005 to 2007. On average, in 2007, more than one third of the overall population seemed to be interested in the possibility of renewing a prescription, scheduling an appointment, or asking their doctors health questions online. This suggests that an attractive market for health organizations using the Internet as part of their business models is developing in Europe. Further research is needed to find out if European citizens would be willing to pay for access to such services, as has been reported in studies conducted in the United States [22].

A pilot study conducted in Poland and Greece showed that three out of four Greek patients (73.2%), once they felt comfortable with telemedicine (28% of the general population), were willing to pay €10 for each online consultation. The percentage of Polish respondents comfortable with telemedicine (35.5%) that were willing to pay for remote medical consultation was found to be somewhat lower (58.3%) but still significant. Among the general population in Poland and in Greece, 1 in 5 respondents said they were willing to pay €10 for a medical online consultation [51].

The situation in Denmark was shown to be remarkable compared with that in the other countries and deserves special mention. Not only was use of the Internet for renewing prescriptions, scheduling appointments, and asking health professionals about health questions reported to be higher, but Danes stood out as having had the highest expectations regarding the possibility of using particular eHealth services in the near future.

Five major factors seem to facilitate a positive environment for eHealth services: regulation/legal framework; reimbursement schemes; security and trust/infrastructure; the eHealth portal; and maturity in Internet adoption and usage.

In Denmark, the collective agreement (CA) between the General Practitioners Association and Danish Regions, via The Regions Salary and Fee Committee (RLTN), specifically addressed and set the terms for electronic communication in general practitioner practices. The CA stated that electronic communication (appointment booking, repeat prescription, and email consultation) must be offered to clinic patients by no later than January 1, 2009, although it was possible to apply for dispensation of the rule. Email consultation must be of a simple, concrete, and nonurgent nature without the need for further information. Lab results can also be offered via email after agreement with the patient. Email consultations are to be reimbursed by a fixed fee. Appointment booking and repeat prescriptions are considered a general service and are not reimbursed.

Security requirements have been established in Denmark. For a patient, there are two ways of sending an email to the doctor: through the eHealth portal sundhed.dk [52], which requires a digital signature, or through the doctor’s own website, which requires logging in. In both cases the email is written on a Web interface where it is Secure Sockets Layer (SSL) encrypted. It is actually converted to an EDIFACT, so it goes directly into the doctor’s information system. The patient receives an ordinary email announcing that there is now a reply to the email from the doctor, and the patient can then log in again either through sundhed.dk or the doctor’s own website to read the message.

Sundhed.dk [52] is the official Danish eHealth portal for the public Danish health care services (*sundhed* means “health” in Danish) that have been brought together on the Internet by the portal. This makes it possible for patients, their families, and health care professionals to access information and to communicate with each other. Use is registered and logged for security. Access to the restricted area (based on proof of employment) is possible for all health care professionals (eg, general practitioners, specialist doctors, dentists, physiotherapists, psychologists, and chiropractors) and organizations (eg, hospitals, pharmacies, and municipalities). Organizations such as municipalities can register for the digital signature as a single unit and assign staff approved by the authorities to it.

Regulatory and technical conditions are echoed by the maturity of the Danish in Internet adaptation and usage [31]. Many daily activities both private and work-related normally take place on the Internet, including public administration. Therefore, using it to communicate with the health care sector is a natural consequence. Secondly, thanks to sundhed.dk, Danes are now familiar with health services and information available online. A survey on the use of electronic services in general practitioner clinics published in December 2007 [53] also clearly showed the same pattern, reporting an increase of more than 75% from 2006 to 2007. However, in the public health care sector, development of electronic patient health record systems has been slow due to the level of decentralized decision-making regarding ICT investment. Pressure for interoperability and the structural reform taking place have either delayed or put many ICT projects in eHealth on hold [54].

### Characteristics of Survey Participants Who Value eHealth Services When Choosing a New Doctor

From the results of logistic regression analysis, we know that, at least for now, the most prevailing characteristics of European citizens for whom availability of eHealth services are important in their decision when choosing a new doctor are age and having completed higher education. This profile changes slightly for those interested in the possibility of scheduling or changing an appointment online to include being a student and living in a main city, while for those interested in communicating by email with the physician, the profile includes being employed. Many reasons may be invoked to explain these findings. For example, younger people may feel more at ease with technology and have a natural predisposition to test new solutions, and people with higher education are likely to have more access to technology. The shortage of time experienced by most professionals may also explain their desire to communicate with their physicians by email.

For the subsample of health-related Internet users, the most interesting findings are the difference in the impact of age on each dependent variable and the interest shown by those having completed secondary school education. That is, the possibility of requesting or renewing prescriptions via email or the Web was attractive to young people up to 25 years old, the possibility of scheduling or changing an appointment online appealed most to those in the 26 to 35 age group, and the possibility of communicating by email--probably a more engaging and demanding activity in terms of responsibility, appealed most to those between 36 and 45 years old. Regarding level of education, we found that among those with access to the Internet, citizens with secondary school education seemed more eager for eHealth services than those with higher education. This finding probably reflects the greater difficulty people with lower levels of education have in reconciling work and visits to the doctor and their lower economic power to pay for health care. This interesting line of research deserves more thorough study in the future. Place of residence also had an influence on these profiles, with those living in big cities giving more value to the possibility of requesting or renewing prescriptions via email or the Web and the possibility of scheduling or changing an appointment online, while there was an indication that communicating by email with the doctor was more appealing to those living in villages. It seems that those who may have easier access to health care appreciate the Internet for simplifying administrative tasks, while those for whom personal contact with health professionals is more difficult because of the distance are more likely to appreciate email communication.

As we could not find any other reported studies of use of the same eHealth services, benchmarking of our results can be done only to a limited extent. Compared with our findings, a study conducted in 2005 [30] showed that online American women were more likely than men to have communicated with a health care provider through the Internet; education was not associated with online patient-provider communication in the multivariate model, age was not a predictor of behaviour, and use of online patient-provider communication was higher among Internet users experiencing health problems or with significant medical histories. A Dutch study of nonusers of e-consultations [32] showed that the elderly, less-educated individuals, chronic medication users, and frequent GP visitors were more motivated to use the service. Several studies have found that online women were more likely than men to search for health information [31,55,56]. However, besides differences at the demographic level, we did not find evidence of higher interest in the eHealth services we studied among those feeling in poor health, those suffering from a disability or long-term illness, or those associated with someone close who is disabled or suffering from long-term illness. In fact, having no disability was found to be a predictor of interest in the possibility of scheduling appointments online.

### Study Contributions and Limitations

The study reported here is novel both in its aim and dimension.

To our knowledge, by the time of the second survey, no reports were available in the literature regarding the extent to which attempts being made by governments in Europe to implement eHealth services were reaching the population at large. Nor was it known the extent to which citizens and even health providers were conscious of what was technically or legally feasible. In fact, recent work confirms that this is still the case in other settings [19]. Therefore, this work represents a timely assessment of conditions being experienced by citizens in the seven countries and of how they perceived and internalized what they knew about efforts being made.

Results are based on representative samples of the populations in seven European countries. In these countries there has been a lack of empirical evidence regarding citizens’ attitudes and expectations towards online interaction with known health professionals and the present levels of use of specific eHealth services. However, the surveys did not cover all European countries. In addition, the possibility of generalizing the results may be hindered by the survey response rate.

## Multimedia Appendix 1

Frequencies of age groups by gender and of sociodemographic variables per country, in 2007

## Multimedia Appendix 2

Observed frequency and percentage of citizens who have requested or renewed prescription via email or web in 2007 in each country and changes from 2005 to 2007

## Multimedia Appendix 3

Observed frequency and percentage of citizens who have scheduled an appointment via the Internet in 2007 in each country and changes from 2005 to 2007

## Multimedia Appendix 4

Observed frequency and percentage of citizens who have asked a particular health question to their family doctor, specialist or other health professional via the Internet in 2007 in each country and changes from 2005 to 2007

## Multimedia Appendix 5

Observed frequency and percentage of citizens expecting to have consultations with health professional online in the year following the survey in 2007 in each country and changes from 2005 to 2007

## Multimedia Appendix 6

Observed frequency and percentage of citizens expecting to schedule an appointment online in the year following the survey in 2007 in each country and changes from 2005 to 2007

## Multimedia Appendix 7

Characteristics of European citizens prizing eHealth services when choosing a new doctor in 2007

## Abbreviations

CATI: Computer-Assisted Telephone Interviews
EHR: Electronic Health Records
ESS: European Social Survey
ICT: Information and Communication Technology
HINTS: Health Information National Trends Survey
SSL: Secure Sockets Layer
WHO: World Health Organization
