# Supplementary figures and images for: Online Communication Between Doctors and Patients in Europe: Status and Perspectives
Source: J Med Internet Res. 2010 Jun 15;12(2):e20. doi: 10.2196/jmir.1281 (PMC2956231; doi:10.2196/jmir.1281)

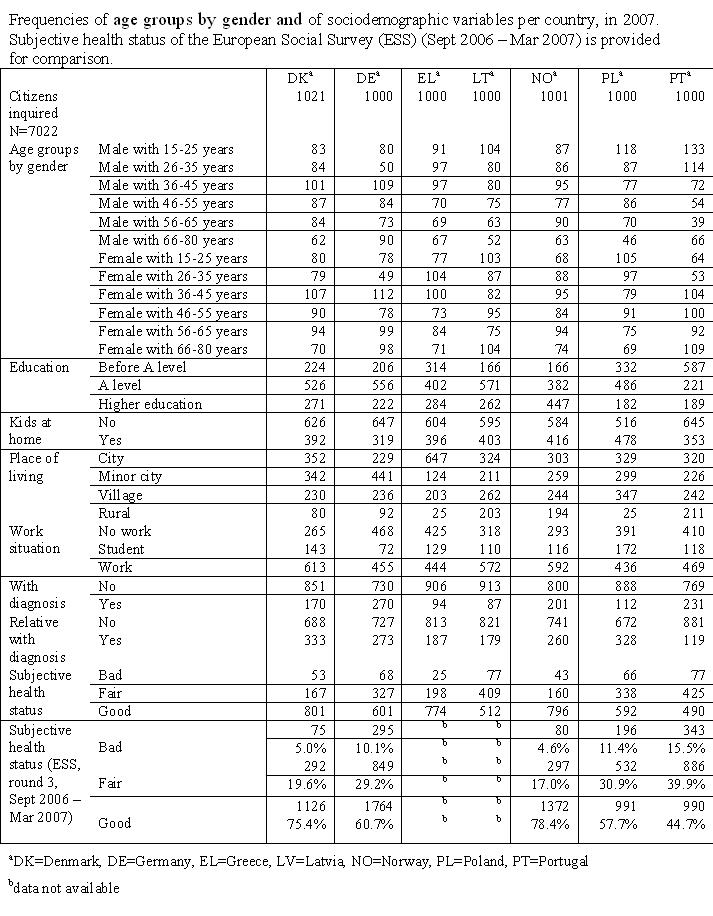

Supplement: Supplementary file 1 [file jmir_v12i2e20_app1.JPG]

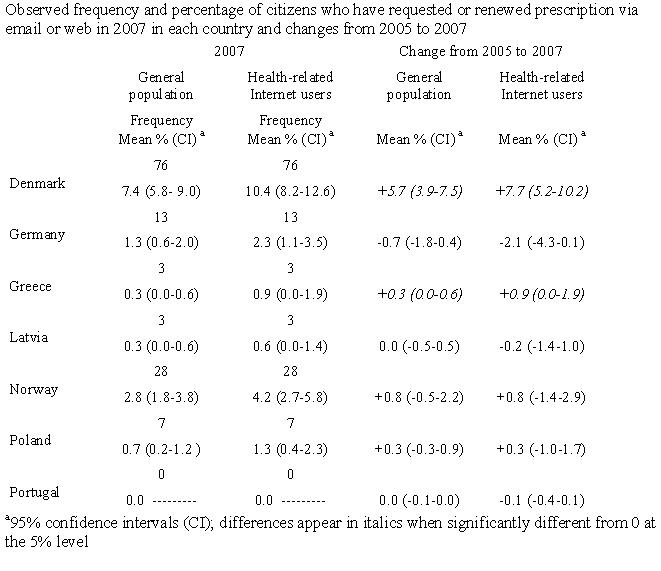

Supplement: Supplementary file 2 [file jmir_v12i2e20_app2.JPG]

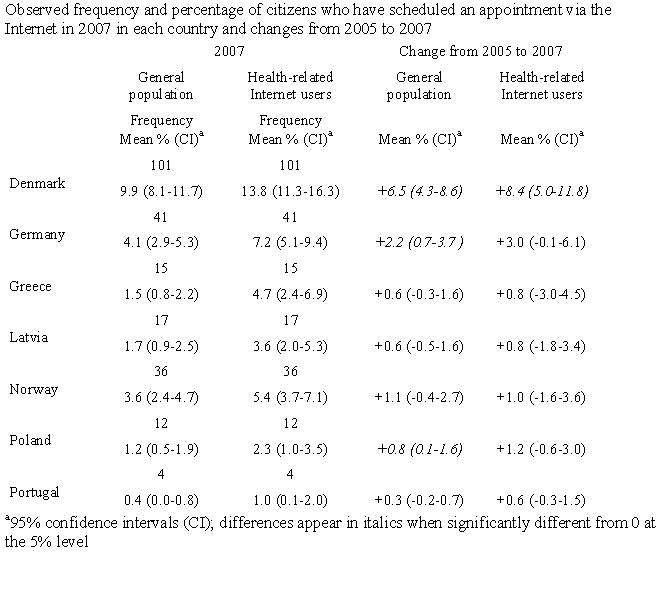

Supplement: Supplementary file 3 [file jmir_v12i2e20_app3.JPG]

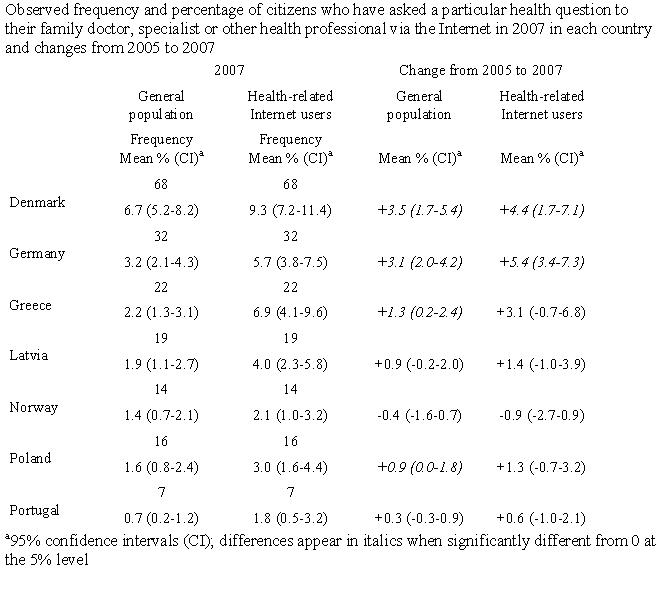

Supplement: Supplementary file 4 [file jmir_v12i2e20_app4.JPG]

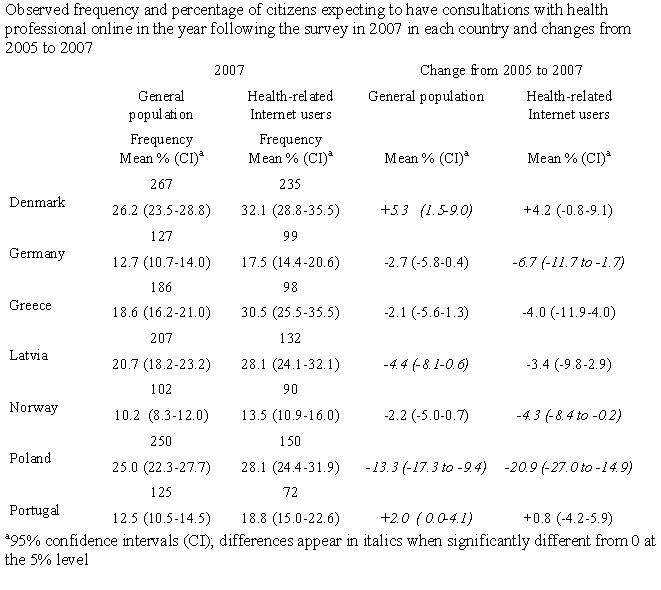

Supplement: Supplementary file 5 [file jmir_v12i2e20_app5.JPG]

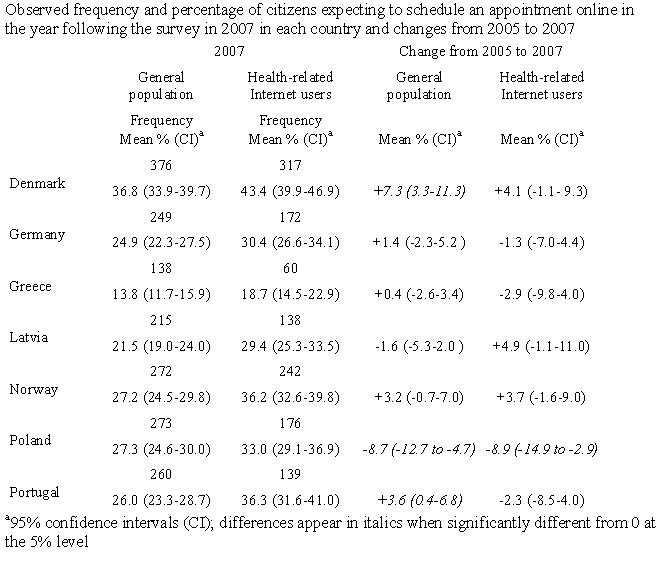

Supplement: Supplementary file 6 [file jmir_v12i2e20_app6.JPG]

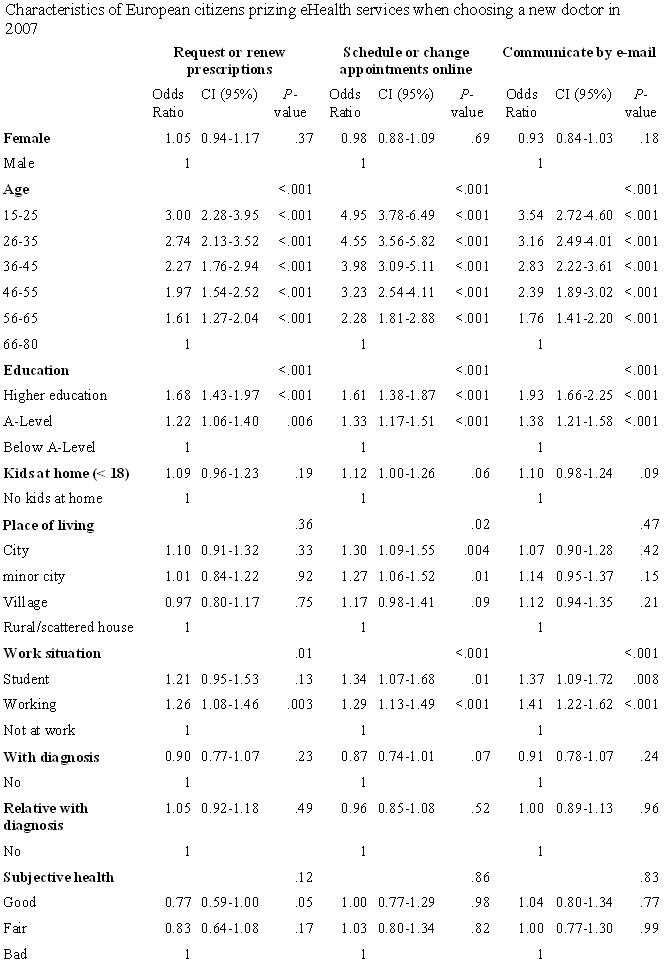

Supplement: Supplementary file 7 [file jmir_v12i2e20_app7.JPG]
